# Response Surface Optimization of a Rapid Ultrasound-Assisted Extraction Method for Simultaneous Determination of Tetracycline Antibiotics in Manure

**DOI:** 10.1155/2015/290903

**Published:** 2015-04-01

**Authors:** Lanqing Li, Mingxing Sun, Hui Zhou, Yun Zhou, Ping Chen, Hong Min, Guoqing Shen

**Affiliations:** ^1^Department of Environment and Resource, School of Agriculture and Biology, Shanghai Jiao Tong University, Shanghai 200240, China; ^2^Shanghai Entry-Exit Inspection and Quarantine Bureau, Shanghai 200135, China

## Abstract

A rapid and cleanup-free ultrasound-assisted extraction method is proposed for the simultaneous extraction of oxytetracycline, tetracycline, chlortetracycline, and doxycycline in manure. The analytes were determined using high-performance liquid chromatography with ultraviolet detector. The influence of several variables on the efficiency of the extraction procedure was investigated by single-factor experiments. The temperature, pH, and amount of extraction solution were selected for optimization experiment using response surface methodology. The calibration curves showed good linearity (*R* ^2^ > 0.99) for all analytes in the range of 0.1–20 *μ*g/mL. The four antibiotics were successfully extracted from manure with recoveries ranging from 81.89 to 92.42% and good reproducibility (RSD, <4.06%) under optimal conditions, which include 50 mL of McIlvaine buffer extraction solution (pH 7.15) mixed with 1 g of manure sample, extraction temperature of 40°C, extraction time of 10 min, and three extraction cycles. Method quantification limits of 1.75–2.32 mg/kg were obtained for the studied compounds. The proposed procedure demonstrated clear reductions in extraction time and elimination of cleanup steps. Finally, the applicability to tetracyclines antibiotics determination in real samples was evaluated through the successful determination of four target analytes in swine, cow manure, and mixture of animal manure with inorganic fertilizer.

## 1. Introduction

Antibiotics are frequently used in veterinary practice to treat and prevent microbial infections [1]. Extensive use of veterinary antibiotics in livestock farming, however, has promoted the development of antibiotic resistance in farm environments. Manure has become a reservoir of resistant bacteria and antibiotic compounds [2]. Little is known about the environmental behavior and fate of antibiotics in manure after cropland application because of analytical difficulties and time-consuming procedures encountered when trying to analyze trace levels of these compounds in the presence of complex matrices such as manure [3]. Therefore, developing a rapid analytical method for the quantification of the most important antibiotics in manure is of great importance.

Tetracyclines (TCs) are an extremely important group of antibiotics with a broad spectrum of activity against Gram-positive and Gram-negative bacteria [4]. All TCs consist of four fused cyclic rings (Table 1). Oxytetracycline (OTC), tetracycline (TC), chlortetracycline (CTC), and doxycycline (DC) are the four main TCs considered as growth-promoting agents and prophylactics for food-producing animals [5]. Because of relatively poor absorption by the gastrointestinal tract, around 25–75% or even 70–90% of the TCs administrated to animals are excreted in their active form [6]. These drugs are released into the environment via urine and feces and can be available for uptake by existing plants [7–9]. Current trends in TCs analysis in the environment focus on food, tissue, or aqueous samples [10, 11]. Unfortunately, quantitation of TCs in animal manure is more difficult than that in these samples. Although detection of TCs can be accomplished by liquid chromatography coupled with mass spectrometry, the use of this methodology requires costly instrumentation that is not always available in routine laboratory analyses, and applications to complex matrices such as manure do not always give good results [8, 9]. Thus, development of sample pretreatment methods with simpler and less sophisticated methodologies is an urgent necessity.

Sample pretreatment is one of the main problems related to the determination of antibiotics in manure because of matrix interference effects [12]. Sample pretreatment methods often include liquid-liquid extraction followed by solid phase extraction (SPE), but methods without SPE have also been proposed [13]. Regardless of the SPE approach selected, the ability of TCs to associate with the sorbent through multiple interactions must be considered [12]. A few studies on simultaneous determination of TCs in manure have been reported, and low and high recoveries have been obtained. Hu et al. [14] and Zheng et al. [15], for example, reported recoveries of 20–81% and 23.3–155.2%, respectively, for simultaneous determination of OTC, TC, and CTC with SPE cleanup; the variability observed in these studies may be explained by SPE losses (breakthrough) or matrix suppression effects [16]. Tylová et al. (2010) described an assay without SPE cleanup for determining four TC antibiotics (i.e., TC, OTC, CTC, and DC) and their epimers in liquid hog manure [17]. However, this simple and direct approach did not yield satisfactory results for CT (recovery, 52.4–72.4%). To the best of our knowledge, no rapid and cleanup-free extraction method yielding satisfactory recovery results for TCs in manure has yet been reported.

Ultrasound-assisted extraction (UAE) is considered a rapid technique that requires only small amounts of solution and inexpensive instrumentation [18]; this method has been successfully applied to antibiotic determination in food and soil [19, 20]. However, several factors, including sonication time, temperature, solution volume, number of sonication cycles, and pH, can influence the UAE process individually and collectively; as such, singling out main independent variables to optimize the extraction process is difficult [21, 22]. Conventional multivariable optimization is usually based on the “one-factor-at-a-time” approach, which is unable to detect interactions among independent variables and presents a lack of complete information on the effects of all determinants [21]. Response surface methodology (RSM) is a useful tool for evaluating multiple parameters and their interactions based on quantitative data that may effectively overcome the drawbacks of classic “one-factor-at-a-time” or “full-factors” approaches [18].

The aim of this study is to develop a simple but effective extraction method for simultaneous determination of four TCs residues in animal manure through HPLC. Here, UAE conditions were investigated. Factors including ultrasonic temperature, solution volume, and pH were optimized by using RSM. The optimal experimental parameters were validated by real samples.

## 2. Materials and Methods

### 2.1. Reagents and Materials

HPLC-grade methanol (MeOH) and acetonitrile (MeCN) were obtained from Merck (Darmstadt, Germany). Distilled water was purified to ultrapure water in a Milli-Q system (Millipore, USA). OTC, TC, CTC, and DC were purchased from Dr. Ehrenstorfer (Augsburg, Germany). All other chemical reagents were of analytical grade and obtained from Sinopharm Chemical Reagent Co., Ltd. (Shanghai, China).

The McIlvaine buffer used in this work was composed of 0.2 M citric acid and 0.4 M Na_2_HPO_4_ (3 : 2, v/v). A mixed solution consisting of McIlvaine buffer, 0.1 M Na_2_EDTA solution, and MeOH at a ratio of 25 : 25 : 50 (v/v/v) was selected as the extraction buffer, similar to previous studies [23–27]. pH was adjusted by addition of H_3_PO_4_ or NaOH.

### 2.2. Standard Solutions and Samples

Individual stock solutions (2.0 mg/mL) were prepared by dissolving each TCs standard in MeOH. Working mixed standard solutions were prepared by mixing appropriate amounts of the four TCs solutions above to obtain a concentration of 500 *μ*g/mL and diluting with MeOH to 100 *μ*g/mL. These solutions were stored in amber glass bottles at 4°C. Cow manure samples were supplied by Agro-tech Extension Center in Pudong District, Shanghai. After air-drying and grinding, samples were sieved to <1 mm before further use.

### 2.3. Extraction of TCs

TCs were extracted using a slightly modified technique [28]. One gram of manure was placed in a 50 mL centrifuge tube and mixed with the extraction buffer. After homogenization, the tube was placed in an ultrasonic bath (Branson B5500S-DTH, Shanghai, China). The sonication time, temperature, solution volume, number of sonication cycles, and pH were set according to the requirements of the experiment. The extract was then centrifuged (Thermo Scientific SL 16 Centrifuge) at 3600 rpm for 2 min. The supernatant was decanted into a new tube and extraction was repeated two times with 20 and 10 mL of extraction buffer. After extraction, all of the manure extracts were combined and centrifuged at 3600 rpm for 5 min.

### 2.4. HPLC Analysis

TCs were determined by a PerkinElmer series 200 HPLC system. Separations were performed on a PerkinElmer SPP C_18_ 2.7 *μ*m, 4.6 × 100 mm column at 30°C, with an injection volume of 20 *μ*L. The UV was set to 355 nm. MeCN, 0.01 M oxalic acid in water, and MeOH were used as the mobile phase and pumped at a rate of 1.0 mL/min. The gradient program began with MeCN : 0.01 M oxalic acid : MeOH = 8 : 84 : 8 (v/v/v) from 0 min to 5 min. This solution was changed linearly to 15 : 70 : 15 (v/v/v) within 1 min, maintained for 6 min, and then returned linearly to initial conditions within 1 min. Equilibration was then performed from 13–20 min at 8 : 84 : 8. All solutions were filtered through 0.22 *μ*m filters (hydrophilic PTFE syringe filter, Anpel, Shanghai, China) before injection into the HPLC system. External calibration curves were constructed by diluting working standard solutions with blank sample extracts to six known concentrations of 0.1, 1, 2, 5, 10, and 20 *μ*g/mL.

### 2.5. Single-Factor Experiments

Single-factor experiments were performed to examine the effects of ultrasonication time (10, 20, 30, 40, and 50 min), number of extraction cycles (1, 2, 3, 4, and 5, resp.), amount of extraction solution (20, 30, 40, 50, and 60 mL, resp.), pH of extraction solution (4, 5, 6, 7, and 8, resp.), and extraction temperature (20, 30, 40, 50, and 60°C, resp.) on extraction efficiency. The independent effect of each factor was determined by changing this factor while keeping all other factors constant. All experiments were performed in triplicate with overnight-spiked samples. To analyze the experimental data, Statistical Analysis System 9.3.1 was employed. Data were declared significantly different when *P* values were lower than 0.05. The ranges of the factors studied were determined by RSM according to the results of the experiments.

### 2.6. Optimization Experimental Design

Based on the results of single-factor experiments, pH (*X*_1_), amount of extraction solution (*X*_2_), and temperature (*X*_3_) were selected as variables for testing in 15-run BBD experiments (Table 2). To optimize the response variable *Y*, to find a suitable approximation for the true functional relationship between independent variables and the response surface is required. The second-order equation is an empirical model and is widely used in RSM [29]. The equation for three factors is as follows:

(1) $$\begin{array}{c} Y=A_{0}+\overset{n}{\underset{i=1}{\sum }}A_{i}X_{i}+\overset{n}{\underset{i=1}{\sum }}A_{ii}X_{i}^{2}+\overset{n}{\underset{i,j=1(i\neq j)}{\sum }}A_{ij}X_{i}X_{j}, \end{array}$$

where *Y* is the response value predicted by the model; *A*_0_, *A*_*i*_, *A*_*ii*_, and *A*_*ij*_ represent the coefficients of the linear, quadratic, and interactive terms, respectively; *X*_*i*_ and *X*_*j*_ are independent variables. Design-Expert 8.0.5 was used to carry out analyses of the experimental design and data, as well as plotting the response surface graphs. Models and regression coefficients were considered significant when *P* values were lower than 0.05.

## 3. Results and Discussions

### 3.1. Optimization of Chromatographic Conditions

TCs spectra show strong absorptions near 275 and 355 nm in neutral and acidic solutions [30]. Therefore, detection wavelengths of 275 and 355 nm were used to monitor TCs in the extraction solution. The latter was selected for further experimentation because of the flatter baseline and sharper peaks resulting from detection at this wavelength. A flow rate of 1.0 mL/min and column temperature of 30°C were determined in consideration of the proper elution time and column pressure.

Different columns, including PerkinElmer Spheri-5 RP C_18_, PerkinElmer SPP C_18_, Pinnacle ODS, and Agilent Athena C_18_ columns, were tested in this study. Under the same chromatographic conditions, only the PerkinElmer SPP C_18_ and Agilent Athena C_18_ columns were able to separate all four analytes. Since a more stable baseline and better peak signal could be obtained by using PerkinElmer SPP C_18_, this column was used for subsequent experiments.

TCs tend to be absorbed on silanol groups in a reversed-phase column to form chelated complexes with metal ions, which lead to peak tailing. As an ionization suppression agent, oxalic acid is usually capable of mitigating the effects of residual silanol on the stationary phase and potentially removing residual metals [12]. Zhou et al. (2009) demonstrated that 0.01 M oxalic acid in pure water can be added to the mobile phase [31]. Viñas et al. (2004) reported that higher pH values and proportion of organic solvent decreased the retention factors of TCs. However, OTC and TC presented a very different retention behavior compared to CTC and DC [32]. Hence, gradient elution was applied in this research and optimized by changing the percentage of oxalic acid. After several trials, the gradient program described in Section 2 was confirmed as the best combination.

### 3.2. Influence of Ultrasound Conditions on TCs Recovery 

#### 3.2.1. Ultrasonication Time

The effect of ultrasonication time on the extraction efficiency of TCs was investigated by varying the ultrasonication time. The results showed that the extraction efficiency of TCs remained relatively constant as ultrasonication time increased from 10 to 50 min, expected for CTC (data not shown). The slightly decreased CTC recoveries observed can be explained by differences between the molecular structures of this antibiotic and other TCs. Additional chlorine atoms on the aromatic ring endow CTC with vulnerability to oxidants, such as ^·^OH, which is produced during ultrasonic irradiation; this vulnerability results in irrelatively higher removal rates of CTC than other TCs [33]. No significant differences were found in the single-factor experiments, which agree with a previous report [34]. Failure to increase recoveries by prolonging the extraction time implies that extraction of TCs from manure is controlled by distribution coefficients rather than kinetics of the desorption process [35]. Manipulation time and extraction costs are expected to increase with increasing ultrasonication time. Thus, the ultrasonication time was set to 10 min in subsequent experiments.

#### 3.2.2. Number of Extraction Cycles

**Figure 1:**
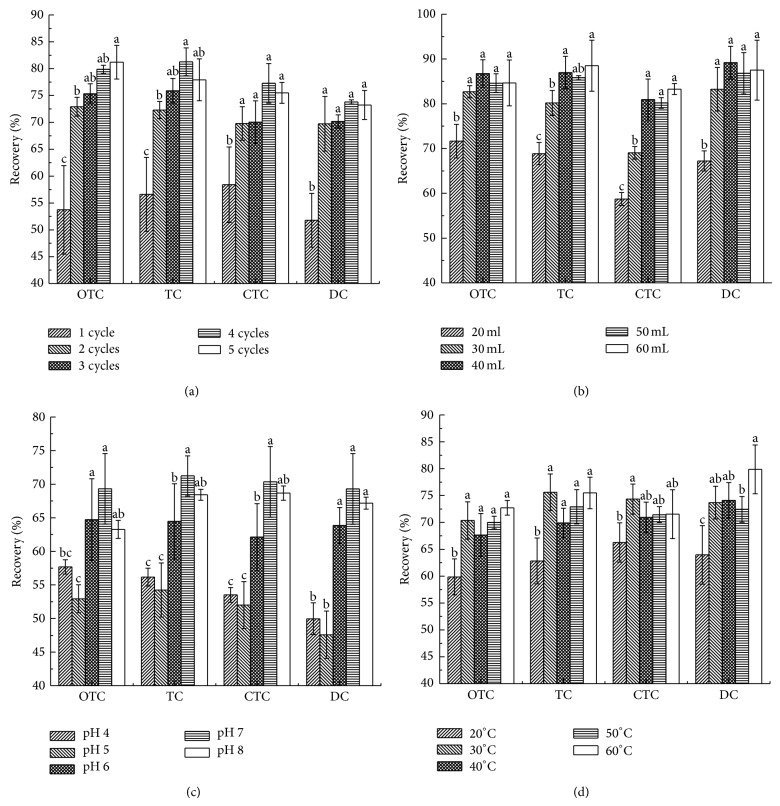
Effects of number of extraction cycles (a), solution volume (b), pH (c), and temperature (d) on OTC, TC, CTC, and DC extraction efficiency (*n* = 3).

As shown in Figure 1(a), increasing the number of extraction cycles increased TCs recoveries, although slight decreases in TC, CTC, and DC were observed when the number of cycles exceeded four. More than one extraction cycle allows introduction of fresh solvent to maintain new equilibrium between the solution and the sample, thereby improving partitioning into the liquid phase [36]. No significant difference in recoveries was observed from extraction cycles between three and five times. Manipulation steps and extraction costs are expected to increase with increasing number of extraction cycles. Therefore, the number of extraction cycles was set to three.

#### 3.2.3. Extraction Solution Volumes

As can be seen from Figure 1(b), TCs recoveries increased significantly with increasing extraction solution volume. Maximum recoveries of 81%–89% were obtained when the solution volume was 40 mL for OTC and DC and 60 mL for TC and CTC. No increase in TCs recoveries was observed with further increases in solution volume. Solution volume obviously exerts an important influence on TCs recovery, which agrees with previous research [31]. Therefore, extraction solution volumes ranging from 30 mL to 50 mL were selected for subsequent optimization.

#### 3.2.4. Extraction Buffer pH

Similar to observations on solution volume, TCs recoveries increased significantly with increasing extraction buffer pH. Maximum recoveries of 69–71% were obtained when the pH of the extraction buffer was 7; recoveries then declined at pH 8. Extraction buffer pH showed a remarkable influence on TCs extraction (Figure 1(c)). While the EDTA-McIlvaine buffer system at pH 4 is the medium used for most TCs extractions from food, some researchers have also reported improved TCs recoveries from soil by adjusting the pH to 7 [3]. In the present study, TCs recoveries at pH of 4 and 5 were significantly lower than those obtained at pH of 6 to 8; this result suggests that the optimal pH is significantly related to the nature of the matrix. Therefore, extraction buffer pH values ranging from 6 to 8 were selected for subsequent optimization.

#### 3.2.5. Extraction Temperature

Figure 1(d) demonstrates that OTC, TC, and CTC recoveries increased obviously from 60–66% to 70–75% as extraction temperature increased from 20°C to 30°C, decreased at a temperature of 40°C, and then increased slightly at higher temperatures. DC showed a different trend: recoveries increased from 20°C to 40°C, decreased slightly, and then finally reached maximum values at 60°C. Except for DC, extraction efficiencies for the drugs generally showed no significant differences at temperatures of 30–60°C. As such, temperatures of 20, 30, and 40°C were selected as the three levels for subsequent optimization experiments.

#### 3.2.6. Optimization by RSM

Based on the results of single-factor experiments, the effects of pH (*X*_1_), volume (*X*_2_) of extraction buffer, and temperature (*X*_3_) on extraction efficiency were determined by RSM; the corresponding ranges and TCs recoveries obtained from these ranges are listed in Table 2. The second-order polynomial model relating the response variable and test factors is as follows:

(2) Y=80.96+1.40X1+4.11X2+3.87X3−2.17X12 −1.67X22+5.24X32+0.55X1X2 −1.31X1X3−0.0568X3.

Analysis of variance was used to evaluate the significance of each factor and interaction terms (Table 3). The coefficient of determination (*R* ^2^) and adjusted coefficient of determination (Adj *R* ^2^) were 0.9889 and 0.9691, respectively, which reveals a good relationship between the actual data and fitted model as well as the high potential of the model to predict responses. The model was significant with a *P* value less than 0.001, and the lack of fit *P* value (0.2540) suggested the excellent applicability of the model. All three independent parameters and quadratic terms significantly affected recoveries; the interactive effect of pH and temperature (*X*_1_ and *X*_3_) on the response was also significant.

**Figure 2:**
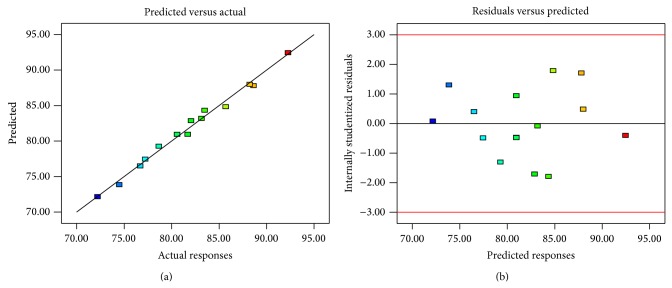
Observed versus predicted responses (a) and internally studentized residuals versus predicted responses (b).

Figure 2(a) shows the predicted versus actual responses. Most of the points were scattered monotonously around the fitting line, which indicates good correlation between the predicted and actual responses.  Figure 2(b) shows the residuals versus predicted responses. The residual points were scattered randomly; therefore, the variance of the experimental measurements is constant for all values of *Y*.

**Figure 3:**
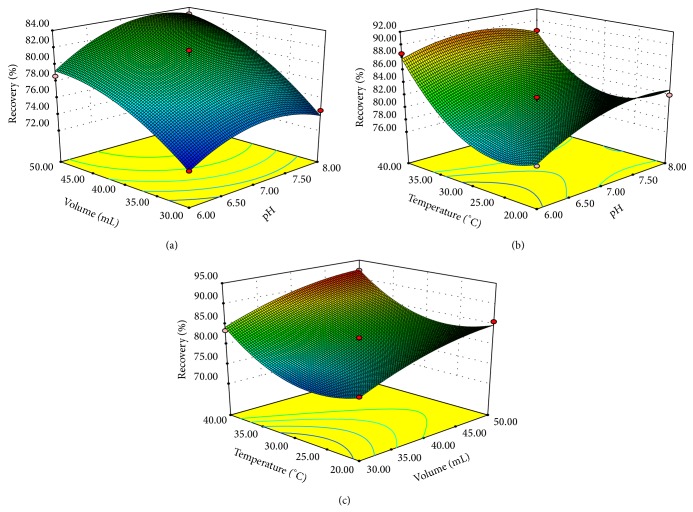
Response surfaces using the Box-Behnken design obtained by plotting: (a) solution pH versus solution volume (extraction temperature: 30°C), (b) solution pH versus extraction temperature (solution volume: 40 mL), and (c) solution volume versus extraction temperature (solution pH: 7).

In order to illustrate the relationship between variables, the response surface graphs were plotted.  Figure 3 depicts interactions between two variables when the third variable is held at zero level for TCs extraction. The combined effect of solution pH and volume is illustrated in Figure 3(a). At a fixed solution volume, extraction efficiency increased as pH increased from 6.0 to 7.4 and then decreased with further increases in pH. The solution volume exerted a positive effect on extraction efficiency, with the response increasing as the buffer amount increased. Figures 3(b) and 3(c) show the response surface obtained by plotting temperature versus pH and buffer volume, respectively. The interaction of pH and temperature was significant (*P* < 0.05). However, the interactive effects of buffer volume and temperature were not significant (*P* > 0.05) (Table 3).

The optimum extraction condition was determined by the ridge maximum analysis. Ridge analysis generates the estimated ridge of maximum response for increasing radii from the center of original design. The ridge maximum analysis predicted that the conditions of 40°C temperature, 7.15 extraction solution pH, and 50 mL volume would lead to the maximum TCs recoveries.

#### 3.2.7. Method Validation

**Figure 4:**
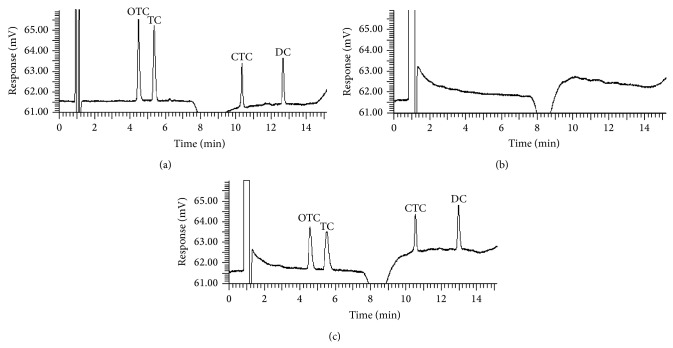
HPLC chromatograms obtained by UV detection (355 nm) of (a) standards of 1.0 *μ*g/mL TCs in pure water, (b) blank manure sample extract, and (c) blank manure spiked with 50 *μ*g/g TCs.

The method validation was performed using spiked samples which were prepared by adding 50 *μ*g/g of TCs to blank manure sample.  Figure 4 presents chromatograms of four TCs obtained from spiked manure sample after UAE; the results of standard solution and blank manure sample are also shown. No interference was detected from endogenous peaks of OTC, TC, CTC, and DC at their respective retention times in blank manure. To evaluate the proposed method, linearity, LOD, LOQ, recovery, and repeatability were investigated under optimized experimental conditions; relevant results are listed in Table 4. Good linearity was obtained for all analytes (*R* ^2^ > 0.9932) in the concentration range of 0.1–20 *μ*g/mL. TCs recoveries were assessed by comparing the amount of analytes added to blank manure samples with the concentrations obtained after extraction. Repeatability was expressed as RSD and calculated from five replicate extractions for one manure sample. Table 4 demonstrates that the recoveries of four TCs ranged from 81.89 to 92.42% at the concentration of 50 *μ*g/g. These values are well within the United States Environmental Protection Agency (US EPA) recommended range of 70–120% [37]. The RSD for each antibiotic was between 2.94% and 4.06%. LOD and LOQ were determined as the lowest concentrations achievable at signal-to-noise ratios of 3 and 10, respectively. The LODs and LOQs for OTC, TC, and DC were 0.03 and 0.1 *μ*g/mL, respectively, while those for CTC were 0.05 and 0.17 *μ*g/mL, respectively. Method quantification limit (MQL) determination was performed according to the US EPA method that uses the variability of multiple analyses obtained from residue-free manure spiked with the four TCs [38]. The MQLs for OTC, TC, CTC, and DC were 1.75, 1.95, 2.32, and 2.15 mg/kg, respectively. Although the maximum residue limits (MRLs) of TCs in animal manure have not been established, the MRLs for all food-producing species were set from 2 to 12 mg/kg in the United States [31]. Therefore, these results confirm the validity of the methodology and its ability to simultaneously determine TCs concentrations in manure.

#### 3.2.8. Application for Real Sample Analysis

The applicability, accuracy, and repeatability of the proposed method were evaluated using ten real fertilizer samples from a local market. None of these samples showed contamination at detectable level, except for one sample with 16.37 mg/kg of CTC. Therefore, in order to determine the accuracy, the relative recoveries were investigated by spiking the fertilizer samples at three concentration levels. The results of spiking three real samples extracted through the proposed method are summarized in Table 5. It can be seen from this table that the recovery values for the analytes range from 71.11% to 116.38%, with RSD < 4.94%. This shows that the proposed procedure is qualified for the analysis of TCs from organic fertilizer.

## 4. Conclusions

In this paper, a simple and rapid sample preparation method for simultaneous detection of four TCs in manure was developed for the first time; this method is based on UAE and coupled with HPLC-UV determination. Single-factor experiments, BBD, and RSM were applied to optimize extraction parameters. According to the results from single-factor experiments, pH, volume of extraction buffer, and temperature were selected to evaluate the interaction and quadratic effects of the variables. Optimal conditions included an extraction solution volume of 50 mL, pH 7.15, temperature of 40°C, ultrasonication time of 10 min, and three extraction cycles. Good recoveries (81.89–92.42%) and RSDs (<4.06%) were obtained, and MQLs ranged from 1.75 mg/kg to 2.35 mg/kg. The method was successfully applied to simultaneously determine four TCs in real manure samples. Therefore, the results of the present work help establish a simpler and more convenient method for simultaneously determining TCs in manure.

## Figures and Tables

**Table 1:** Hydrophobicity, p*K*_*a*_ values, and structures of four TCs.

		p*K*_*a*_	log⁡*K*_ow_
Oxytetracycline (OTC)	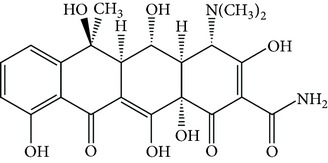	3.22; 7.46; 8.94	−0.890

Tetracycline (TC)	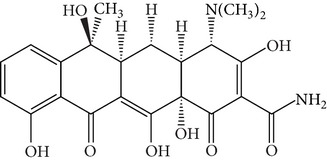	3.32; 7.78; 9.58	−1.131

Chlortetracycline (CTC)	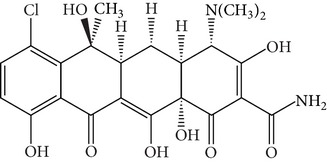	3.33; 7.55; 9.33	−0.360

Doxycycline (DC)	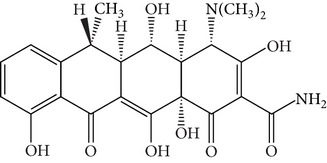	3.02; 7.97; 9.15	−0.024

**Table 2:** Box-Behnken design with actual/coded values and results of tetracycline antibiotics recovery.

	*X*_1_ pH	*X*_2_ volume (mL)	*X*_3_ temperature (°C)	*Y* recovery (%)
1	6 (−1)	30 (−1)	30 (0)	72.20
2	8 (+1)	30 (−1)	30 (0)	74.49
3	6 (−1)	50 (+1)	30 (0)	78.65
4	8 (+1)	50 (+1)	30 (0)	83.15
5	6 (−1)	40 (0)	20 (−1)	77.22
6	8 (+1)	40 (0)	20 (−1)	82.05
7	6 (−1)	40 (0)	40 (+1)	88.63
8	8 (+1)	40 (0)	40 (+1)	88.23
9	7 (0)	30 (−1)	20 (−1)	76.69
10	7 (0)	50 (+1)	20 (−1)	85.70
11	7 (0)	30 (−1)	40 (+1)	83.48
12	7 (0)	50 (+1)	40 (+1)	92.26
13	7 (0)	40 (0)	30 (0)	80.59
14	7 (0)	40 (0)	30 (0)	81.70
15	7 (0)	40 (0)	30 (0)	80.58

**Table 3:** Analysis of variance of the response surface quadratic model.

Source	Sum of squares	Df^a^	Mean square	*F*-value^b^	*P* value^c^ Prob > *F*
Model	418.10	9	46.46	49.74	0.0002
*X*_1_, pH	15.74	1	15.74	16.85	0.0093
*X*_2_, volume (mL)	135.3	1	135.3	144.86	<0.0001
*X*_3_, temperature (°C)	119.66	1	119.66	128.11	<0.0001
*X*_1_ *X*_2_	1.22	1	1.22	1.31	0.3047
*X*_1_ *X*_3_	6.84	1	6.84	7.32	0.0425
*X*_2_ *X*_3_	0.013	1	0.013	0.014	0.9099
*X*_1_^2^	17.34	1	17.34	18.57	0.0077
*X*_2_^2^	10.26	1	10.26	10.99	0.0211
*X*_3_^2^	101.49	1	101.49	108.67	0.0001
Residual	4.67	5	0.93		
Lack of fit	3.84	3	1.28	3.09	0.2540
Pure error	0.83	2	0.41		
*R* ^2^	0.9889				
Adj *R* ^2^	0.9691				

^a^Degree of freedom.

^b^Test for comparing model variance with residual (error) variance.

^c^Probability of seeing the observed *F*-value if the null hypothesis is true.

**Table 4:** Figures of merit for TCs using the proposed UAE^∗^.

TCs	Linear regression equation	*R* ^2^	LOD *μ*g/mL	LOQ *μ*g/mL	MQL mg/kg	Recovery%	RSD%
OTC	*Y* = 2.839 × 10^5^ *X* − 2.749 × 10^4^	0.996650	0.03	0.10	1.75	92.42	2.94
TC	*Y* = 3.239 × 10^5^ *X* − 2.834 × 10^4^	0.996960	0.03	0.10	1.95	87.85	3.88
CTC	*Y* = 1.673 × 10^5^ *X* − 6.695 × 10^3^	0.996559	0.05	0.17	2.32	81.89	4.06
DC	*Y* = 2.371 × 10^5^ *X* − 3.190 × 10^4^	0.993216	0.03	0.10	2.15	84.46	2.37

^∗^Recoveries and RSD were obtained at the concentration of 50 *μ*g/g.

**Table 5:** The related recoveries of TCs in fertilizer samples by proposed method.

TCs	Added (mg/kg)	SM			CM			MIM		
		Found (mg/kg)	Recovery (%)	RSD (%)	Found (mg/kg)	Recovery (%)	RSD (%)	Found (mg/kg)	Recovery (%)	RSD (%)
OTC	5	4.09	81.75	2.95	3.65	73.00	0.21	5.75	114.93	0.54
	25	21.41	85.64	4.09	23.37	93.50	3.12	27.06	108.23	2.96
	100	83.61	83.61	0.18	95.09	95.09	0.28	102.09	102.09	1.11

TC	5	3.86	77.12	0.83	3.88	77.61	3.65	5.10	102.00	4.87
	25	21.53	86.1	3.62	24.13	96.51	3.05	26.92	107.69	0.28
	100	80.64	80.64	0.39	90.14	90.14	0.23	103.27	103.27	0.95

CTC	5	4.10	81.95	1.48	3.82	76.30	2.11	4.90	98.09	4.22
	25	25.24	100.97	2.02	21.00	84.02	0.12	25.36	101.44	1.03
	100	85.68	85.68	1.30	82.46	82.46	0.01	102.07	102.07	2.93

DC	5	4.16	83.26	1.25	3.56	71.11	2.97	5.82	116.38	4.94
	25	20.18	80.7	0.57	21.76	87.04	0.25	28.22	112.90	3.88
	100	87.12	87.12	0.74	86.65	86.65	1.40	103.10	103.10	4.38

Note: SM: swine manure; CM: cow manure; MIM: mixture of inorganic fertilizer with manure.
